# Shoulder and Elbow Surgery Special Issue

**DOI:** 10.1111/os.12813

**Published:** 2020-11-16

**Authors:** Marius M Scarlat, Yong Cheng Hu

**Affiliations:** ^1^ Clinique Chirurgicale St Michel, Groupe ELSAN Toulon France; ^2^ International Orthopaedics, Official Journal of the SICOT Brussels Belgium; ^3^ Tianjin Hospital Tianjin China; ^4^ Editorial Office of “Orthopaedic Surgery” Tianjin China

China is a great country, and history teaches us that it was the largest producer and provider of goods and tools, as well as being a nation that developed many concepts and ideas in different fields of activity, including medical science.

Medical communication and journals are used to share knowledge, and it took us centuries to learn that the key to success is the quality of the teaching and the results. Outcome studies and peer‐review processes changed our way of pursuing medical careers and libraries, and databases are now the depositaries of great knowledge.

The development of shoulder surgery followed this algorithm in all medical specialties. However, traditional Chinese medicine had solutions for the treatment of shoulder pain, muscular contractures, weakness, and other symptoms and syndromes related to patient discomfort and quality of life. A recent systematic review outlined the use of Tuina as a traditional medicine method in the treatment of the frozen shoulder. Tuina is the use of a certain part of the hand or limb by a physician on a patient to press, push, grasp, roll, and pinch, producing a biological effect and eventually improving clinical symptoms^1^. Acupuncture, too, is widely used in China and also in many other countries to treat different conjunctive tissue disorders, including shoulder contractures, stiffness, and pain^2^.

Modern shoulder surgery was developed in the Western world by Bankart and Codman. The vision of shoulder pathologies was further enlightened by Charles Neer, who was the founder of the modern specialty of shoulder surgery based on anatomical knowledge, outcome studies of the surgery for rotator cuff pathologies, arthroplasty, instabilities, and various traumatic and degenerative conditions that were treated traditionally in general surgery services or with conservative methods. Neer's teachings were followed and upgraded by his illustrious fellows from North America and Europe, including Frederick A. Matsen from Seattle and Charles A. Rockwood from San Antonio, the authors of the major monograph and textbook *The Shoulder*, which is widely used and currently at its fifth edition, containing the work of contributors from many countries. The European School of Shoulder and Elbow Surgery brought important contributions to the development of the specialty, including the works of Latarjet and the Lyon School proudly represented by Gilles Walch and his followers, the Dijon School of Prof. Grammont, inventor of the modern reverse shoulder arthroplasty, the Swiss School of N. Schwengt and Christian Gerber, and many other national schools and institutes that developed shoulder and elbow knowledge in Europe and worldwide. Many shoulder and elbow schools have Chinese fellows and colleagues who are actively contributing to the scientific development of shoulder and elbow surgery in China. Scientific publications in the field of shoulder and elbow surgery include many valuable Chinese contributors and respected authors, and this special issue is an example of the volume and quality of research and clinical work performed in China.

The predecessors of Chinese orthopaedic surgeons, including professors Feng Chuanhan, Guo Shiba, and Huang Gongyi, published *Shoulder Surgery* – a text which outlined the development of this specialty in China^3^.

The academic environment for shoulder and elbow surgery is very good and the International Congress of Shoulder and Elbow Surgery is held every year. Teaching and training meetings and workshops are organized by regional central hospitals. In May 2014, the Chinese Shoulder and Elbow Society (CSES) was established with Prof. Jiang Baoguo from the Peking University People's Hospital elected as first chairman^4^. The Chinese Journal of Shoulder and Elbow was founded in November 2013 and distributed electronically. The current chairman of the CSES is Prof. Chunyan Jiang, a well‐known scientist and respected shoulder surgeon.

The current special issue of *Orthopaedic Surgery* includes scientific papers from different shoulder, elbow, and orthopaedic departments that specialize in articular surgery and quality modern treatment.

It is with pleasure and pride that the French group of surgeons led by Prof. Philippe Hernigou from Paris presents the paper concerning the osteonecrosis of the humeral head, based on a very important clinical experience^5^.

Recent advances in the arthroscopic management of shoulder pathologies are presented by teams from Shanghai^6^ and Guagzhou^7^.

Traumatology is well‐represented and specific specialized solutions are described by highly qualified colleagues from different centers in Quingdao, Shanghai, Tianjin, and Chengdu^8^, ^9^, ^10^, ^11^. Technical improvements and solutions in humeral nailing are described by Xiao‐ming Wu from Shanghai^12^. Traumatology is an important part of the shoulder and upper limb specialty, and the trauma services are constantly developing techniques and procedures dedicated to better patient care, minimally invasive procedures, and lower radiation exposure during the procedure, which is better for the surgical team and also for the patient.

Shoulder anterior instability and treatment based on anatomic repair and reconstruction are an important part of our specialty. All major shoulder services are performing different arthroscopic and open techniques of reconstruction based on accurate evaluation of the sources of instability and good quality ligamentar, capsular, and bone repair^13^, ^14^, ^15^, ^16^, ^17^.

Reverse shoulder arthroplasty became very popular in the last 20 years, and currently China is developing this chapter for better patient care, offering new solutions in shoulder reconstruction for orthopaedic and traumatic conditions. A recent work was published in Chinese by Bo Lu from Shizhajuang^18^, and two papers included in this special issue concern traumatic and rheumatoid destructive conditions treated by reconstruction with reverse shoulder arthroplasty^19^, ^20^.

The subacromial space and management of cuff conditions are explored in two papers by Chengdu and Xiamen^21^, ^22^.

The elbow specialty is developed either in upper limb services or in combination with shoulder services. Chinese elbow specialists are extremely proficient and publish excellent results on both traumatic and orthopaedic elbow conditions. In this special issue, the elbow traumatology includes papers from Beijing, Chengdu, and Tianjin^23^, ^24^, ^25^, ^26^. The excellent elbow arthroscopy papers are authored by Jiuzhou Lu and colleagues from Shanghai. They describe experience with the techniques of release for post‐traumatic elbow contracture and stiffness and with heterotopic ossifications occurring in the elbow after arthroscopic release^27^, ^28^.

The publication of a special issue of *Orthopaedic Surgery* dedicated to the shoulder and elbow specialty is an event that requires readers' attention. Neglected in the past and reduced to some basic procedures for many years when lower limb surgery or trauma were the focus of publications, shoulder and elbow surgery has now become a modern part of orthopaedics, performed currently with high‐quality technical tools, outstanding optical devices, arthroscopes, image intensifiers, fluoroscopes, high‐frequency ultrasound machines, radiology or computer tomography scan devices used preoperatively. The final beneficiary is the patient that will eventually experience shorter hospital stay, minimally invasive surgery, insignificant or reduced bleeding, solid repairs, early mobilization and shorter medical leave, earlier return to work or usual activities, and overall better quality of life.

## References

[1] Ai J , Dong YK , Tian QD , Wang CL , Fang M . Tuina for periarthritis of shoulder; a systematic review protocol. Medicine (Baltimore), 2020, 99: e19332 10.1097/MD.0000000000019332. 32176055 PMC7220300

[2] Yang C , Lv TT , Yu TY , Wong S , Lu MQ , Li YZ . Acupuncture at Tiaokou (ST38) for shoulder adhesive capsulitis: what strengths does it have? A systematic review and meta‐analysis of randomized controlled trials. Evid Based Complement Alternat Med, 2018, 2018: 4197659 10.1155/2018/4197659. 29849707 PMC5937513

[3] Feng CH , Guo SF , Huang GY , *et al* Shoulder Surgery, 1st edn Tianjin: *Tianjin Science and Technology Publishing House*, 1996.

[4] Jiang BG . Shoulder and elbow surgery in China. Chin Med J (Engl), 2014, 127: 3841 10.3760/cma.j.issn.0366-6999.20142240. 25421176

[5] Hernigou P , Hernigou J , Scarlat M . Shoulder osteonecrosis: pathogenesis, causes, clinical evaluation, imaging, classification. Orthop Surg, 2020, 12: 1340–1349. 10.1111/os.12788 PMC7670135 33015963

[6] Sun Q , Ge W , Li G , *et al* Plate fixation versus arthroscopic‐assisted plate fixation for isolated medium‐sized fractures of the greater tuberosity: a retrospective study. Orthop Surg, 2020, 12: 1456–1463. 10.1111/os.12773 PMC7670132 33073535

[7] Alike Y , Hou JY , Tang YY , *et al* Arthroscopic superior capsular reconstruction incorporated with rotator cuff repair to restore static and dynamic stabilizer of shoulder: an integrated repairment for a massive irreparable rotator cuff tear. Orthop Surg, 2020, 12: 1503–1510. 10.1111/os.12768 PMC7670152 32851772

[8] Liu T , Bao FL , Jiang T , Ji GW , Li JM , Jerosch J . Acromioclavicular joint separation: repair through suture anchors for coracoclavicular ligament and nonabsorbable suture fixation for acromioclavicular joint. Orthop Surg, 2020, 12: 1362–1371. 10.1111/os.12771 PMC7670157 32893498

[9] Zhang L , He AN , Jin YF , Cheng HW . Novel double endobutton with aguiding locator: a biomechanical study of reconstruction in acromioclavicular joint dislocation. Orthop Surg, 2020, 12: 1511–1519. 10.1111/os.12770 PMC7670143 32812693

[10] Wu K , Wu XM , Zha XL , Wang QG . Anatomic restoration of triple disruption of the superior shoulder suspensory complex: a case report and review of the literature. Orthop Surg, 2020, 12: 1526–1530. 10.1111/os.12764 PMC7670163 32975039

[11] Zhang Q , Xiang M , Li YP , Yang JS . Arthroscopic management of glenoid and greater tuberosity bipolar fractures: clinical and radiological outcomes. Orthop Surg, 2020, 12: 1405–1412. 10.1111/os.12786 PMC7670139 33078582

[12] Fu HC , Wang F , Wang QG , Wu XM . Reduced surgical time in intramedullary nailing for humeral shaft fracture without radiation exposure when using electromagnetic targeting system. Orthop Surg, 2020, 12: 1413–1420. 10.1111/os.12785 PMC7670153 32893489

[13] Yang JS , Xiang M , Chen H , Li YP , Zhang Q , Dai F . Risk factors of graft resorption after arthroscopic autologous scapular spine bone graft for recurrent shoulder instability: does general factors play a role in graft resorption? Orthop Surg, 2020, 12: 1388–1393. 10.1111/os.12778 PMC7670148 33200578

[14] Yang YL , Li QH , Zhang Q , *et al* Treatment of chronic anterior shoulder dislocation or subluxation by coracoid osteotomy with or without Bristow‐Latarjet procedure. Orthop Surg, 2020, 12: 1478–1488. 10.1111/os.12776 PMC7670138 32975042

[15] Dai F , Xiang M , Yang JS , *et al* The injury mechanism of acute anterior shoulder dislocation associated with glenoid and greater tuberosity fracture: a study based on fracture morphology. Orthop Surg, 2020, 12: 1421–1429. 10.1111/os.12767 PMC7670144 32812705

[16] Wang Y , Zhou ZY , Zhang YL , *et al* Early follow‐up of arthroscopic latarjet procedure with screw fixation and suture‐button fixation for recurrent anterior shoulder instability. Orthop Surg, 2020, 12: 1350–1361. 10.1111/os.12781 PMC7670134 33200576

[17] Lyu F , Wang HX , Bi C , Shen SM , Wang QG , Wu XM . Management of dislocation of the shoulder joint with ipsilateral humeral shaft fracture: initial experience. Orthop Surg, 2020, 12: 1430–1438. 10.1111/os.12782 PMC7670156 32812708

[18] Lu B , Scarlat MM . Reverse shoulder arthroplasty: current trend in elective surgery. Zhong Hua Gu Ke Za Zhi, 2018, 38: 627–634.

[19] Tian X , Xiang M , Wang GY , *et al* Early evaluation of the treatment of complex proximal humeral fractures in the elderly with reverse shoulder arthroplasty. Orthop Surg, 2020, 12: 1372–1379. 10.1111/os.12777 PMC7670161 33015981

[20] He Y , Xiao LB , Zhai WT , Xu YL . Reverse shoulder arthroplasty in patients with rheumatoid arthritis: early outcomes, pitfalls and challenges. Orthop Surg, 2020, 12: 1380–1387. 10.1111/os.12769 PMC7670150 32803918

[21] Zeng YM , Xu C , Zhang K , Yu DG , Zhang J . Can acromial morphology predict the related rotator cuff injury? A three‐dimensional measurement study. Orthop Surg, 2020, 12: 1394–1404. 10.1111/os.12774 PMC7670155 33200577

[22] Zhuo HW , Zhu FG , Pan L , Li J . The use of autologous iliotibial band with Gerdy's tubercle for the management of irreparable rotator cuff tears. Orthop Surg, 2020, 12: 1489–1494. 10.1111/os.12799 PMC7670137 33015994

[23] Zhang HL , Lin KJ , Lu Y , *et al* Can the size of the fragment in comminuted coronoid fracture be predicted by the contralateral side? An analysis of similarity of bilateral ulnar coronoid morphology. Orthop Surg, 2020, 12: 1495–1502. 10.1111/os.12780 PMC7670165 33017086

[24] Liu JY , Zhang JZ , Wang YM , Tian X , Dong JM . Posterior Monteggia fractures or posterior fracture‐dislocation of proximal ulna in adults? Orthop Surg, 2020, 12: 1448–1455. 10.1111/os.12784 PMC7670164 32790243

[25] Teng L , Zhong G . Surgical treatment of comminuted coronal shear fracture of distal humeral bone‐19 cases experience. Orthop Surg, 2020, 12: 1439–1447. 10.1111/os.12765 PMC7670145 32979039

[26] Zhang BX , Zhang H , Zhang Q , *et al* Redirection using double pulley technique for snapping triceps tendon: a case report and technique note. Orthop Surg, 2020, 12: 1520–1525. 10.1111/os.12772 PMC7670127 33200574

[27] Dai JX , Zhang GF , Li SL , Xu JG , Lu JZ . Arthroscopic treatment of posttraumatic elbow stiffness due to soft tissue problem. Orthop Surg, 2020, 12: 1464–1470. 10.1111/os.12787 PMC7670133 33015918

[28] Yang CQ , Hu JS , Xu JG , Lu JZ . Heterotopic ossification after arthroscopic elbow release. Orthop Surg, 2020, 12: 1471–1477. 10.1111/os.12801 PMC7670160 33200575

